# Flexible biofilm monitoring device

**DOI:** 10.1002/elsc.202100076

**Published:** 2022-07-12

**Authors:** Viktoria Maria Reiferth, Dirk Holtmann, Daniela Müller

**Affiliations:** ^1^ Technische Hochschule Mittelhessen University of Applied Sciences Giessen Germany

**Keywords:** biofilm monitoring, biofilm susceptibility testing, biofilms, MBEC, *Pseudomonas*

## Abstract

Biofilms and their analysis are increasingly attracting the attention of the scientific community due to the immense importance and impact of biofilms in various natural, technical and medical fields. For these purposes, an optimized and extended antibiofilm assay system based on the Calgary Biofilm Device (MBEC Assay® system) consisting of microtiter plate and PCR tubes was established. Its implementation was used to study the growth characteristics of the sessile phenotype of *Pseudomonas fluorescens* exposed to antimicrobial peptides. Inhibitory effects of an antimicrobial peptide on *P. fluorescens* biofilm formation could be determined at a concentration of 250 μg/ml (biofilm prevention concentration (BPC)) using the modified biofilm assay. Similarly, the biofilm bactericidal concentration (BBC) at 125 μg/ml and the minimum biofilm elimination concentration to remove 90% of the total biofilm mass (MBEC90) were measured at a concentration range of 15.625–1.95 μg/ml. In conclusion, this optimized system provides a highly variable, simple, and cost‐effective alternative to high‐throughput screening based on the Calgary Biofilm Device (CBD).

**Abbreviations**
BBC: biofilm bactericidal concentration
BIC: biofilm inhibitory concentration
BPC: biofilm prevention concentration
CBD: calgary biofilm device
CFU: colony forming unit
CV: crystal violet
MBC: minimum bactericidal concentration
MBEC: minimum biofilm elimination concentration
MBEC90: minimum biofilm elimination concentration to remove 90% of the total biofilm mass
MBIC: minimum biofilm inhibitory concentration
MIC: minimum inhibitory concentration
OD600: optical density at 600 nm
PP: polypropylene
rpm: rounds per minute

## 1 INTRODUCTION

For instance, formation of biofilms on medical devices, such as catheters or implants often results in difficult‐to‐treat chronic infections [1, 2]. Likewise, biofilm formation causes tremendous problems in various industries, including industrial water systems, oil industry, and process industries. Therefore, identification and optimization of compounds that specifically target biofilm‐forming bacteria is necessary to address a growing challenge, so anti‐biofilm activity of natural and synthetic compounds has attracted great interest. A wide variety of approaches consisting of colorimetric, biochemical, genetic, mass spectrometric, and microscopic techniques can be used for visualization and quantification of biofilms [3, 4, 5]. Considering that each method has advantages and disadvantages associated with the evaluation of limited parameters, it is obvious that, there is no method that allows a complete analysis of the complex biofilm [6]. Similarly important as choosing the right biofilm analysis method is selecting the appropriate in vitro biofilm model obtaining reproducible and reliable results. Several closed systems, with the advantage of simplicity and applicability for high‐throughput analysis, and open systems, which allow better control of nutrient delivery, temperature and flow, have evolved in recent years [7].

A rapid, simple, reproducible, and cost‐effective method is needed to rapidly develop and screen compounds for anti‐biofilm activity. Comparing all common methods, 96‐well scale applications are the most favorable and suitable ones to be used especially in high throughput techniques [8, 9]. Unlike the determination of minimum inhibitory concentration (MIC), where the assay endpoint can be easily determined based on the binary type of “growth versus no growth” [7], the variations in biofilm architecture and the complexity of its maturation complicate the interpretation used to detect and monitor biofilm growth. In general, such assays that stain the remaining attached biofilm biomass after incubation with a compound of interest are referred to as BIC (biofilm inhibitory concentration), MBIC (minimum biofilm inhibitory concentration), MBEC (minimum biofilm eradication concentration), and MBC (minimum bactericidal concentration) assays [6]. The Calgary Biofilm Device (CBD) has been described as a technology for the rapid and reproducible assay of biofilm susceptibilities to antibiotics and is one of the most frequently used in susceptibility testing [10]. In this study a simple and inexpensive alternative to the CBD was developed from polypropylene (PP) microcentrifuge tubes and 96‐well plates.

Polypropylene (PP) is a chemically inert polymer with highly versatile mechanical properties that make it suitable for a wide range of medical devices (e.g., catheters, hernia meshes, and sutures). Therefore, the development of reproducible and cost‐effective in vitro PP models to study biofilms is important.

In this study, we describe a simple polypropylene (PP) biofilm model that can be easily fabricated from materials available in most microbiology laboratories. This model mimics the CBD and is less expensive than a CBD. Our device uses up to 96 PP tubes instead of the pins used in the CBD.

**PRACTICAL APPLICATION**

Unwanted biofilms are a widespread problem in medicine and engineering. In addition to the development of chemicals that act against the biofilms, their characterization also presents a particular challenge. Biofilm resistance to anti‐microbial or anti‐biofilm agents combined with their complex structure and dynamic nature make biofilm formation quite difficult to measure and control. In this technical note an easy and flexible measurement procedure, based on a commercial system, is presented. The portrayed method uses inexpensive and easily available components and can be performed by people without scientific education. A wide variety of surfaces can be examined by simple adaptions. Due to these properties, the described test system can be used in many different applications and research topics in the future.

*Pseudomonas fluorescens* (*P. fluorescens*) is not generally considered a bacterial pathogen in humans; however, multiple culture‐based and culture‐independent studies have identified it at low levels in the indigenous microbiota of various body sites. While significantly less virulent than *P. aeruginosa*,* P. fluorescens* can cause bacteremia in humans, with most reported cases being attributable to either transfusion of contaminated blood products or use of contaminated equipment associated with intravenous infusions [11]. Therefore, this species was chosen for assay development and validation.

## 2 MATERIAL AND METHODS

### 2.1 MBEC using the CBD

The CBD from Innovotech Inc. (Edmonton, Canada) comprises a two‐part reaction vessel. The upper part forms a lid with 96 cones, which is sealed at the top. These pins are designed to be placed into wells of the bottom assembly in the reaction vessel, as well as into wells of a standard 96‐well plate. This bottom component has been designed for channeling medium flow across the pins, creating a homogenous shear force across all pins resulting in formation of uniform biofilms at each pin location.

### 2.2 Design of upgraded MBEC‐assay

#### 2.2.1 Establishment of the antibiofilm assay

**FIGURE 1:**
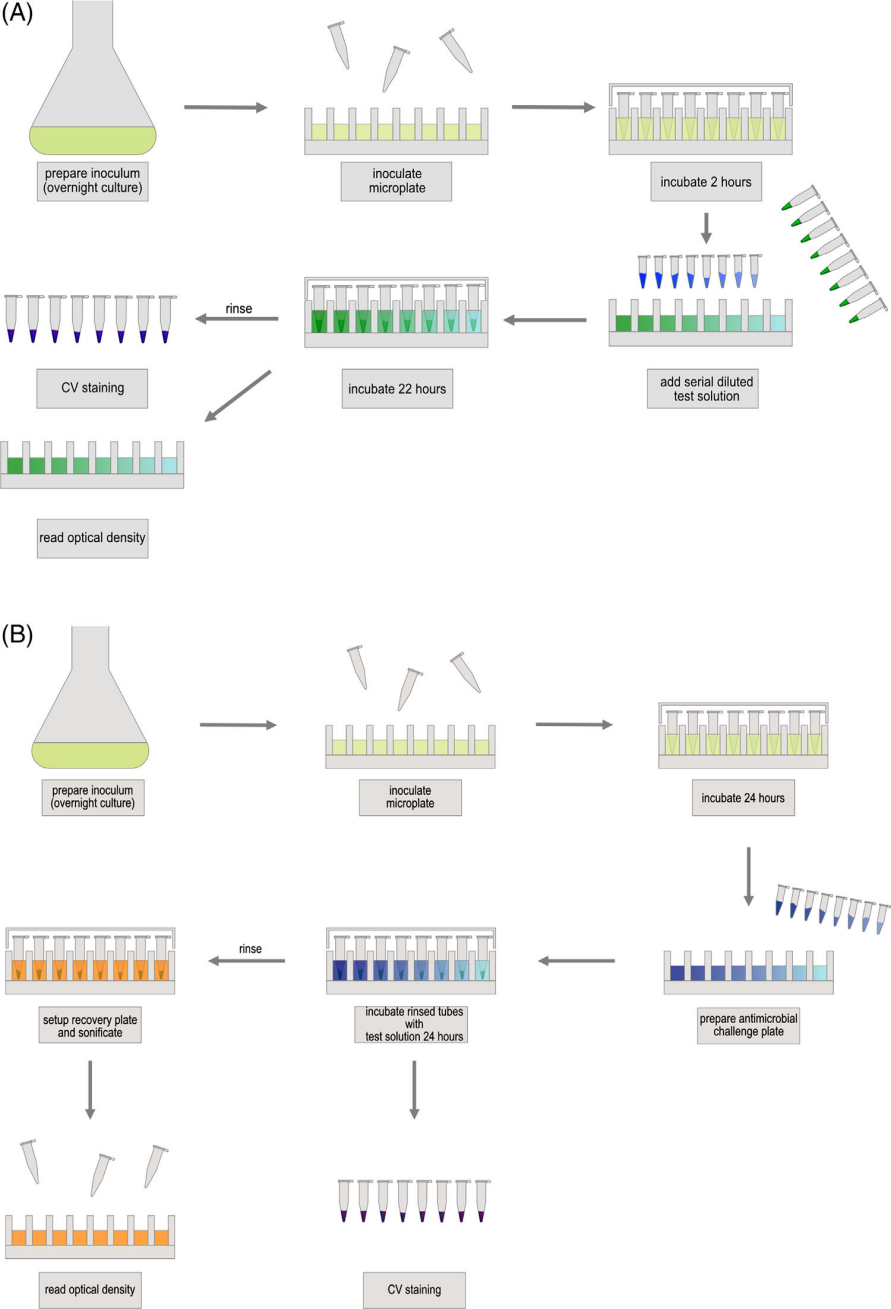
Schematic illustration of PCR‐tube antibiofilm assay: (A) MBIC used for determining the minimal peptide concentration needed to eradicate fully grown biofilm, (B) MBEC used for determining the minimal concentration which is needed to prevent biofilm growth onto the pegs; description see in Section 2.2.3 in the main text above

Based on the CBD an enclosed system of 96‐well microtiter plates and PCR tubes was constructed. Using this system, biofilms of a wide variety of bacteria and microorganisms can be generated by high‐throughput screening for testing efficacy of antimicrobial agents. Additionally, this assay design allows simultaneously testing of several reagents at various concentrations. PCR tubes were inserted into the wells and their external sides are intended to serve as surfaces for bacteria to form biofilms. Avoiding evaporation of the medium and minimizing risk of contamination, the plate lid was attached to the bottom plate by adhesive tape after the PCR tubes were inserted. Schemes of the developed MBEC assay are shown in Figure 1.

In an initial experiment, biofilm formation of *P. fluorescens* DSM‐289 was tested by loading a 96‐well microtiter plate under sterile conditions as shown in Figure 1 by adding 120 μl bacterial suspension from a fresh 24 h overnight culture as well as 120 μl medium acting as sterile control. Inoculum was used from a petri dish stored at 4°C.

Inoculum OD600 was set to 0.01 and controlled by photometric measurement. Autoclaved PCR tubes were individually inserted into each well, sealed with tape, wrapped in plastic wrap, and incubated for 24 h at 30°C and 110 rpm. PCR tubes were removed, washed in 140 μl of 0.9% (w/v) sodium chloride (NaCl) solution removing planktonic cells. Biofilm was fixed in 140 μl of 100% methanol for 5 min and dried overnight. Biofilm was stained with 140 μl of a 0.5% (v/v) crystal violet (CV) solution for 30 min, followed by washing each PCR tube three times in 140 μl sterile MilliQ water and drying overnight. Quantification of total biofilm mass was obtained by detaching the dye attached to the biofilm by 140 μl of a 30 % (v/v) acetic acid solution for 30 min. Absorbance of CV was determined at 570 nm.

#### 2.2.2 Growth curve of sessile phenotype of *P. fluorescens*

Growth characterization of sessile *P. fluorescens* phenotypes, biofilm formation on the PCR tube surface was observed for 48 h. Therefore, an inoculum with OD600 of 0.01 was prepared from an overnight culture by diluting it with LB medium and pipetted into a microtiter plate as shown in Figure 1. After being measured photometrically at 600 nm, the plate was incubated at 30°C, 110 rpm. At various times, four PCR tubes were taken for crystal violet staining and four tubes were taken for colony forming unit (CFU) counting.

CV was used to stain the total biofilm mass as described in Section 2.2.1. For CFU counting, PCR tubes were washed in 140 μl of a 0.9 % (m/v) NaCl solution for 5 min, transferred to 180 μl of fresh NaCl solution, and placed in an ultrasonic bath for 30 min detaching the biofilm of the PCR tube surfaces. Samples were then serial diluted 1:10 and 20 μl of each was plated out on LB agar plates according to Miles and Misra. [12] Klicken Sie hier, um Text einzugeben. After 24 h incubation at room temperature (22–25°C), colonies were counted. Room temperature was sufficient to obtain countable colonies. Otherwise, colonies formed were too large/big.

#### 2.2.3 Determination of bactericidal biofilm concentration (BBC) and minimum biofilm elimination concentration (MBEC) of antimicrobial peptide HH7

The ability of an antimicrobial substance to remove already formed, mature biofilms can be described by the MBEC. MBEC is the lowest concentration of a substance that is required completely removing the total biofilm formed. Another definition of anti‐biofilm activity is BBC. This refers to the concentration that kills 99.9% of all cells embedded in biofilms. This can thus be understood as the minimal bactericidal concentration (MBC) at biofilm level. The MBC defines the concentration of an antibiotic at which just 99.9% of all cells in a planktonic culture are killed within a defined period of time.

Inoculum was prepared as described above. For biofilm formation, subsequently, 120 μl of the inoculum was pipetted into wells 1–11, and 120 μl of the sterile controls into well 12. Again, the microtiter plate was read out photometrically (OD600), and sterile PCR tubes were inserted into each well. The plate was incubated for 24 h at 30°C and 110 rpm allowing biofilm formation.

PCR tubes from A2 to A11 served as biofilm growth controls to quantify the total biofilm using CV. The cell number of the biofilms formed was determined with tubes from H2 to H11 by a CFU assay according to Section 2.2.2.

After incubation, all PCR tubes except these used as biofilm growth controls were removed, washed in 140 μl 0.9% NaCl (w/v) and transferred to the “challenge plate” (Figure 1). This contained the peptide solutions (wells 2–11), sterile controls for medium and peptide (wells 12) and sterile medium for growth controls (wells 1) (all 120 μl). Therefore, a serial dilution of the peptide in MilliQ was prepared. The challenge plate was also measured photometrically before and after incubation. After incubation for additional 24 h at 30°C and 110 rpm, all PCR tubes were washed in 140 μL NaCl (0.9% (w/v)). Half of the tubes were used for CV staining, therefore fixed as described in Section 2.2.1. All remaining PCR tubes were transferred to a new microtiter plate, each containing 120 μl of sterile LB medium, and placed on an ultrasonic bath for 30 min. PCR tubes were removed, optical density (OD600) determined, and incubated at 30°C, 110 rpm until growth controls showed significant turbidity. OD600 was also determined after incubation.

BBC is defined at that peptide concentration where no visible turbidity is seen after incubation (∆OD600 ≤ 0.05). In addition, MBEC was determined based on the removal of the total biofilm mass quantified by CV. The peptide concentration at 100% biofilm removal corresponds to the MBEC.

## 3 RESULTS AND DISCUSSION

An antibiofilm assay was established according to the CBD from Innovotech Inc. This represents a standardized high‐throughput screening method for testing biofilm susceptibility or antimicrobial efficacy against biofilms of a wide variety of microorganisms. It consists of a 96‐well microtiter plate with a bottom composed of 96 individual wells or a corrugated plate that can contain only a single microorganism. On the top plate of the assay system are pegs, available in a variety of materials, which protrude into the wells of the bottom plate. Under batch conditions and gentle shaking, biofilms form on these pegs, which can then be subjected to testing.

Nevertheless, this system, using fixed pegs in 96‐well format, has some limitations, such as evaporation of the medium. To circumvent these limitations or variate surfaces within one plate, an extended, optimized system could be developed and established.

### 3.1 Growth curve of sessile phenotype of *P. fluorescens*

In previous experiments, the parameters start OD600, temperature, rotation per minute (rpm) and incubation time for biofilm formation of *P. fluorescens* were already determined and transferred to the optimized system. As described above, the system was tested at 30°C, 110 rpm for 24 h with a start OD600 of 0.01.

Using the PCR tube system (consisting of PP), homogenous biofilm formation can be observed, which is required for testing on BPC, MBEC, BBC and recording kill kinetics. It was observed, that denser and thicker biofilms were formed at an air‐liquid interface, which can be related to improved aeration. Since quantification of biofilms in these tests is done only at the end of the experiment on CV or CFU determination, an equal total biofilm mass forming must be guaranteed. Assuming a uniform biofilm formation without verification could lead to incorrect results. A misinterpretation is especially given under the false assumption of heterogeneous biofilm formation.

With the newly developed PCR tube system, uniform biofilms could be grown. The sterile controls showed no growth, despite the fact that the lid did not fit exactly with the microtiter plate. This was achieved by sealing the resulting gap with adhesive tape, which also counteracted the risk of evaporation of the medium, which occurred more frequently with the CBD.

Using the optimized system, biofilm formation of the gram‐negative bacterium *P. fluorescens* on the outer surface of the PCR tubes was investigated. Therefore, an inoculum with a start OD600 of 0.01 was prepared as described and placed in a microtiter plate. Subsequently, the PCR tubes on whose surface the biofilms were formed were added. Incubation at 30°C was performed for 48 h with gentle shaking.

**FIGURE 2:**
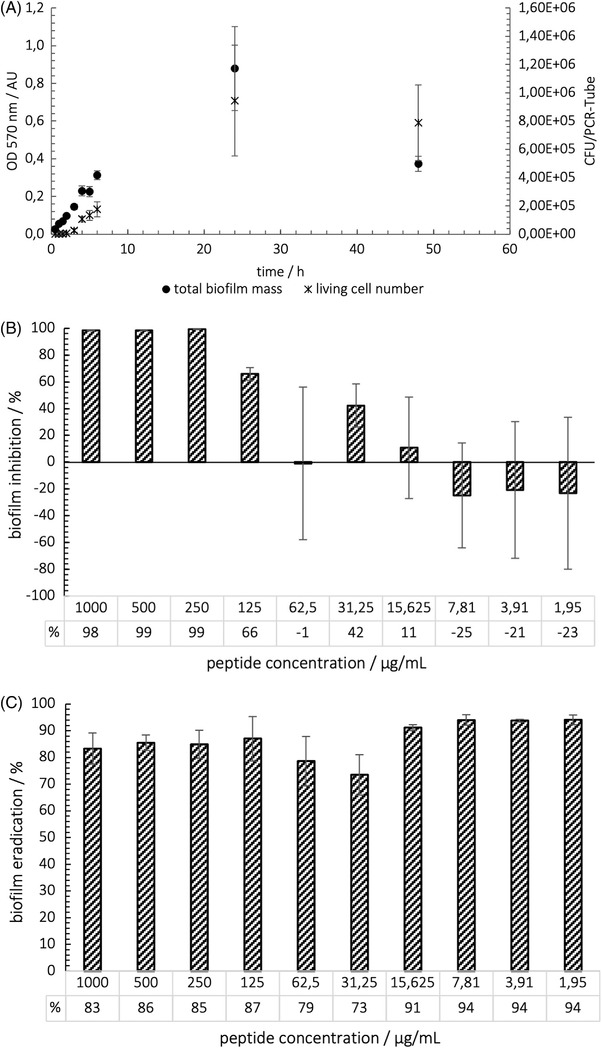
(A) Growth curve of sessile *P. fluorescens* DSM‐289 in LB medium during 48 h at 30°C and 110 rpm (starting OD = 0.01). Optical density determination at 600 nm (*n* = 60) living cell number calculated with CFU‐Assay according to Miles and Misra, (B) MBIC Growth inhibition of *P. fluorescens* biofilm by treatment with different peptide concentrations determined by crystal violet staining (*n* = 6), (C) MBEC: Growth reduction of *P. fluorescens* biofilm induced by several peptide concentrations (*n* = 3)

A differentiation should be done between cells and matrix when studying biofilms in place. Total biofilm mass, including matrix, was quantified by CV staining. Living cell numbers were calculated by CFU determination according to Miles and Misra [12]. Figure 2A represents the variation of total biofilm mass and live cell count of *P. fluorescens* biofilms as a function of time.

The increase in biomass was approximately linear within the first 6 h. There was a further increase in biomass up to 24 h. This value is significantly higher than the previous ones with a measured optical density at 600 nm of 0.88 ± 0.22. Subsequently, a decrease in mass was registered after 48 h of incubation. Here, an OD600 of 0.37 ± 0.04 was obtained, which is slightly higher than the obtained OD600 at 6 h. When looking at the live cell count as a function of incubation time, a different pattern was seen. Within the first hour, no increase in cell number could be determined. Even after another 30 min, only a very small increase could be determined. Only from hour 2 did cell growth begin in the biofilms. From this point on, uniform growth was detectable.

Likewise, the live cell number reached a maximum value of approximately (9.45 ± 3.92) × 10^5^ CFU/PCR tube after 24 h. No reduction in the number of viable cells was observed after 48 h of incubation. Within the calculated standard deviations, there is no difference after 24 and 48 h. The delay in cell growth during the first 1.5 h can be described as a kind of lag phase, in which no increase in viable cells took place, but only an adaptation to changed conditions. Here, these would be described as the phenotypic change of the cells themselves. In the first phase of biofilm formation, the attachment, the initial contact of the cells with the surface to be colonized takes place.

### 3.2 Determination of biofilm prevention concentration (BPC), bactericidal biofilm concentration (BBC) and minimum biofilm elimination concentration (MBEC) of antimicrobial peptide HH7

The BPC describes the concentration required to reduce the viable cell numbers of planktonic cultures in order to prevent biofilm formation. This inhibitory effect of peptide HH7 was studied using the PCR tube system as described. For this, the inoculum was pre‐incubated within the microtiter plate for 2 h, followed by the addition of the diluted peptide solutions. OD600 was measured before and after incubation using a microplate reader to monitor cell growth in suspension (Figure 1B).

We also tested the eradicative effect of the AMP HH7 on mature 24 h old *P. fluorescens* biofilms. For this purpose, *P. fluorescens* biofilms were first formed on PCR tubes, which were then incubated with different concentrations of the peptide for additional 24 h. After incubation, the PCR tubes were rinsed. Half of the tubes were subjected to further incubation to distinguish surviving cells. The biofilms attached to the remaining tubes were stained with CV to detect possible clearance by the peptide.

Inhibitory efficacy of AMP HH7 on *P. fluorescens* biofilm formation was determined by a concentration of 250 μg/ml, called BPC, using the adopted antibiofilm assay (Figure 2B). Equally, the BBC was achieved at 125 μg/ml and the MBEC to eradicate 90% of the total biofilm mass (MBEC90) was achieved at a concentration range of 15.625–1.95 μg/ml (Figure 2C).

## 4 CONCLUDING REMARKS

Investigation of biofilms and their sensitivity to antibiotic substances often relies on closed static systems. The CBD model consists of a 96‐well microtiter plate and an associated top plate with 96 so‐called pegs and allows the formation of 96 equal biofilms. The CBD provides a low‐shear, high‐throughput batch system that is robust, responsive, repeatable, and reproducible. It is ideally suited as a high‐throughput method for screening antimicrobial or antibiofilm substances at different concentrations for biofilm monitoring.

While the CBD device costs 20–36 € (Innovotech Inc.), the system described here costs about 8 €. PP PCR tubes are available for 8 € per 100 (Carl Roth GmbH & CoKG) and microplate are available for 50 € per 100 (Carl Roth GmbH & Co.KG). In addition, this system allows the handling of each biofilm separately without disruption to other biofilms and more important is the reproducibility of the quantitative counts of the biofilms produced in this reactor which make it a promising method for measuring the susceptibility of biofilms to several compounds.

The CBD is available in several versions. In addition to the classic polystyrene version, systems with coated pegs, e.g. with L‐lysine, hydroxyapatite or titanium dioxide, are also available and biofilm formation can be investigated on various abiotic surfaces. Microtiter plates and PCR tubes are usually made of polypropylene, polystyrene, polycarbonate or polyethylene and the surfaces can be easily modified or coated with several chemicals. This may result in different surface properties which can be tested in parallel using our adapted version.

Potential ways to optimize this system include the use of deep‐well plates in combination with PCR tube strips. This would minimize contact between the surface of the microtiter plate and the PCR tubes during incubation. This would have less impact on biofilm formation. Additionally, the tubes could be inserted deeply into the wells so that the PCR tube tip would not rest on the bottom like a standard 96‐well plate and would be surrounded by enough medium that biofilms could form there, too. The use of PCR strips could save a considerable amount of time while allowing variable plate occupancy.

The high flexibility with regard to corresponding surface properties, microorganisms to be tested, test substances used, combined with a high reproducibility, makes the alternative developed here a universally applicable, valid test system in the field of biofilm monitoring.

## CONFLICT OF INTEREST

The authors have declared no conflict of interests.

## Data Availability

Data available on request from the authors.

## References

[1] Rumbaugh KP . How well are we translating biofilm research from bench‐side to bedside? Biofilm. 2020;2:100028. 33447813 10.1016/j.bioflm.2020.100028 PMC7798461

[2] Wi YM , Patel R , Understanding biofilms and novel approaches to the diagnosis, prevention, and treatment of medical device‐associated infections. Infect Dis Clin North Am. 2018;32(4):915‐929. 30241715 10.1016/j.idc.2018.06.009 PMC6215726

[3] Felz S , Vermeulen P , van Loosdrecht MCM , Lin YM . Chemical characterization methods for the analysis of structural extracellular polymeric substances (EPS). Water Res. 2019;157:201‐208. 30953855 10.1016/j.watres.2019.03.068

[4] Allkja J , Bjarnsholt T , Coenye T , et al. Minimum information guideline for spectrophotometric and fluorometric methods to assess biofilm formation in microplates. Biofilm. 2020;2:100010. 33447797 10.1016/j.bioflm.2019.100010 PMC7798448

[5] Pantanella F , Valenti P , Natalizi T , Passeri D , Berlutti F . Analytical techniques to study microbial biofilm on abiotic surfaces: pros and cons of the main techniques currently in use. Ann ig : med prev comunita. 2013;25(1):31‐42. 10.7416/ai.2013.1904 23435778

[6] Azeredo J , Azevedo NF , Briandet R , et al. Critical review on biofilm methods. Crit Rev Microbiol. 2017;43(3):313‐351. 27868469 10.1080/1040841X.2016.1208146

[7] Macià MD , Rojo‐Molinero E , Oliver A . Antimicrobial susceptibility testing in biofilm‐growing bacteria. Clin Microbiol Infect. 2014;20(10):981‐990. 24766583 10.1111/1469-0691.12651

[8] Stiefel P , Rosenberg U , Schneider J , Mauerhofer S , Maniura‐Weber K , Ren Q . Is biofilm removal properly assessed? Comparison of different quantification methods in a 96‐well plate system. Appl Microbiol Biotechnol. 2016;100(9):4135‐4145. 26923144 10.1007/s00253-016-7396-9 PMC4824840

[9] Subramanian S , Huiszoon RC , Chu S , Bentley WE , Ghodssi R . Microsystems for biofilm characterization and sensing ‐ a review. Biofilm. 2020;2:100015. 33447801 10.1016/j.bioflm.2019.100015 PMC7798443

[10] Ceri H , Olson ME , Stremick C , Read RR , Morck D , Buret A . The Calgary Biofilm Device: new technology for rapid determination of antibiotic susceptibilities of bacterial biofilms. J Clin Microbiol. 1999;37(6):1771‐1776. 10325322 10.1128/jcm.37.6.1771-1776.1999 PMC84946

[11] Scales BS , Dickson RP , LiPuma JJ , Huffnagle GB . Microbiology, genomics, and clinical significance of the *Pseudomonas fluorescens* species complex, an unappreciated colonizer of humans. Clin Microbiol Rev. 2014;27(4):927‐948. 25278578 10.1128/CMR.00044-14 PMC4187640

[12] Miles AA , Misra SS , Irwin JO . The estimation of the bactericidal power of the blood. J. Hyg. 1938;38(6):732‐749. 20475467 10.1017/s002217240001158x PMC2199673

